# Some Newer Antibiotics Active Against *Helicobacter pylori* and Anaerobic Bacteria and the Potential Benefits of Their Wider Availability in More Countries: A Narrative Review

**DOI:** 10.3390/antibiotics15060581

**Published:** 2026-06-07

**Authors:** Lyudmila Boyanova, Liliya Yordanova Boyanova, José Medeiros, Georgi Dimitrov, Petyo Hadzhiyski, Raina Gergova, Rumyana Markovska

**Affiliations:** 1 Department of Medical Microbiology, Medical University of Sofia, 2 Zdrave St, 1431 Sofia, Bulgaria; lili_9204@hotmail.com (L.Y.B.); georgi.dimitrov.swy@gmail.com (G.D.); rtgergova@gmail.com (R.G.); markovska73@abv.bg (R.M.); 2 Faculty of Medicine, University of Coimbra, 3000 Coimbra, Portugal; jmedeiros@fmed.uc.pt; 3 Children’s Gastroenterology Clinic, Specialized Hospital for Active Pediatric Treatment “Prof. Dr. Ivan Mitev”, Medical University-Sofia, 1431 Sofia, Bulgaria; dr.hadjiiski@abv.bg

**Keywords:** anaerobes, *Clostridioides difficile*, *Helicobacter pylori*, acne, bacterial vaginosis, periodontal diseases, newer antibiotics, activity, advantages, access

## Abstract

It is crucial to consider newer antibiotics with activity against anaerobes and *Helicobacter pylori*, given their healthcare importance, and the constantly growing antibiotic resistance/multidrug resistance, which complicates the therapy. The aim of this review was to emphasize certain recently approved or still-under-investigation antibiotics with potential benefits for treating *Clostridioides difficile* infections (CDIs), other anaerobic infections, and those caused by *H. pylori*, covering recent data from articles published primarily in 2020–2026. Given the limited number of antibiotics for treating CDI and fidaxomicin nonavailability in many countries, it is necessary to conduct more extensive laboratory and clinical studies of promising antibiotics such as ibezapolstat, delafloxacin, lascufloxacin, omadacycline, eravacycline, ridinilazole, and CRS3123. Against *Bacteroides fragilis* group species, delafloxacin and eravacycline showed good activity. Research on rifasutenizol for bacterial vaginosis, sarecycline and nadifloxacin for acne vulgaris and amixicile for periodontal diseases needs to be expanded. For *H. pylori* infection, delafloxacin, sitafloxacin, nemonoxacin, zoliflodacin, and rifasutenizol may improve the suboptimal success of most eradication regimens. However, more efforts, in coordination between medical, scientific, manufacturing, and government representatives, should ensure wider access to and research on the newer antibacterials. Establishing more research groups, careful examination of market issues, and additional approaches, such as nanomaterials, efflux pump inhibitors, phage therapy, and CRISPR-Cas systems, should be beneficial. Notwithstanding the difficulties, there are many opportunities to promote research on and potential use of newer antibiotics which show advantages over the older antibacterials, and to make them available to numerous countries and patients worldwide.

## 1. Introduction

Many studies focus on the resistance and multidrug resistance (MDR) of facultative anaerobic and obligate aerobic bacteria, as well as the potential benefits of introducing or using newer antibiotics for their treatment, while studies on anaerobic and microaerophilic bacteria are fewer. One reason is that these bacteria are not routinely cultured in all microbiology laboratories, as they require specific incubation conditions, culture media, and interpretation. However, both anaerobes and *H. pylori* (microaerophiles) are bacteria with enormous clinical significance.

The problem of increasing antibiotic resistance and MDR in anaerobes is a topic of high diagnostic, clinical, and therapeutic importance. *Bacteroides fragilis* group (BFG) is widely recognized as one of the most clinically significant and frequently isolated group of anaerobic bacteria in human infections, as well as one of the most virulent and often MDR group of species [1,2].

Carbapenem resistance is caused by class B metallo-beta-lactamases, such as those encoded by BFG *cfiA* genes [2]. Although certain strains are *cfiA*-positive, high-level resistance usually requires an upstream insertion sequence element [2]. BFG isolates have a variety of other resistance determinants, such as MDR efflux pumps, *nimB* (nitroimidazole resistance) genes, and *cepA* (endogenous cephalosporinase) gene, and there are isolates with insertion sequences that were positive for both *cfiA* and *nimB* genes [2,3,4].

Toxigenic *Clostridioides difficile* can cause severe inflammation of the colon, resulting in watery or bloody diarrhea and its most severe form, pseudomembranous colitis, and moreover, up to 30% of the patients suffer from relapsing infections [5]. Hypervirulent strains such as ribotype (RT) 027 (ST1 clone) and RT078 (ST11), or in the hypervirulent ST1 clone, cause the most severe infections and were observed both in hospitals and in the community [6].

More than half of the isolates of *C. difficile* had MDR to antibiotics that may cause CDI, which is typically found in epidemic or hypervirulent strains. Some investigations have shown resistance to the three antibiotics used to treat *C. difficile* infection (CDI)—metronidazole, vancomycin, and fidaxomicin, although resistance to the latter is still absent or low [5].

MDR was also detected in other anaerobes such as *Prevotella* spp. [3]. Newer antibacterials may offer new hope [7,8,9,10], but optimizing the treatment of anaerobic infections by the newer agents requires further evaluation.

As one of the most common causes of human infections and a potentially carcinogenic bacterial species that infects over 40% of the world’s population, *Helicobacter pylori*, has important medical significance [11]. *H. pylori* causes chronic active gastritis, which can lead to peptic ulcers, gastric adenocarcinoma and mucosa-associated lymphoid tissue (MALT) lymphoma in part of the infected subjects [12,13]. To eradicate *H. pylori* infection, many combination regimens have been used; they often consist of acid-decreasing medications (vonoprazan or proton pump inhibitors, PPIs) plus two or three antibacterial drugs, sometimes with the addition of bismuth preparations [12]. Nonetheless, antibiotic resistance in *H. pylori* is rising in many countries, and MDR rates varied from less than 10% to more than 40% [11].

Knowledge of the characteristics of newly approved, investigational, or less known antibiotics and some of their advantages over older agents in the same antibiotic class would be helpful in optimizing the treatment of patients with anaerobic or *H. pylori* infections once these new agents become widely available. In addition, the results may provide directions for future research.

Given the published data concerning (1) the frequency, severity, recurrence rate, and limited treatment options of CDIs, (2) the presence and increasing resistance/MDR of anaerobic bacteria and *Helicobacter pylori* to antibiotics, as well as (3) the wide prevalence of the frequently lifelong *H. pylori* infection linked to peptic ulcers and two malignant tumors [1,2,3,8,11,14,15,16], it is increasingly necessary to take into account newer antibiotics for potential treatment or prevention of the associated diseases.

The aim of this review article was to focus on some less-known or recently approved, or still investigational antibacterial agents with potential benefits for treating anaerobic bacteria, such as *C. difficile*, BFG and other anaerobic species, as well as the microaerophilic species *H. pylori*, with an emphasis on their structure, activity and potential use. Discussions of their success or lack thereof in different stages of clinical trials are also included, along with a variety of associated factors.

## 2. Materials and Methods

We encompassed and discussed data from recent publications using PubMed, Scopus, ScienceDirect, and Google Scholar databases. Our search was conducted between January 2026 and the end of April 2026. The articles included were in English.

According to Boolean search queries, four groups of keywords were used:

(1) Regarding the antimicrobials: (“new” OR “newer” OR “novel” OR “first-in-class”) AND (“antibiotic” OR “antimicrobial” OR “derivative” OR “candidate” OR “approved” OR “investigational”).
(2) As for the bacteria: (“*Clostridioides difficile*” OR “*Bacteroides*” OR “*Cutibacterium acnes*” OR “periodontal” OR “*Helicobacter pylori*”) AND (“anaerobes” OR “anaerobic”, OR “microaerophilic” OR “microbiota”).
(3) Relating to the studies: (“FDA-approved” OR “multicenter” OR “multicentre”) AND (“study” OR “trial” OR “meta-analysis”), NOT (“congress abstract”, NOT “conference paper”).
(4) Relating to the availability of antibiotics: (“availability” OR “access”) AND (“Europe” OR “developing countries”) AND (“strategy” OR “approach)”.

Criteria for inclusion and exclusion.

A criterion for inclusion was the selection of newer antibiotics based on the availability of publications related to their activity against clinically significant anaerobic and microaerophilic bacteria for which there is a need to optimize therapy and counteract existing antibiotic resistance. For this purpose, we considered their advantages, such as antibiotic susceptibility, safety profiles compared to older representatives of the same antibiotic class, clinical trials if they were available, and therapeutic success. Other criteria for inclusion were publication dates, preferably since 2020, and the language of the articles in English. The criteria for exclusion were the data from congress abstracts and conference papers.

The criteria used to define an antibiotic as “newer” or “recently approved” were to select antibacterial agents approved by the FDA (The U.S. Food and Drug Administration), the EMA (The European Medicines Agency), or agencies in other countries for the treatment of MDR infections, although not specifically for anaerobes and *H. pylori*, as well as investigational antibiotics, depending on whether recent studies on the activity of these agents against anaerobes and microaerophiles were available.

Our aim was to compile data on the activity of newer antibiotics against the anaerobic and microaerophilic bacteria, given their importance to public health and the need to improve the treatment of the associated infections. Since it is not possible to cover all newer antibiotics, this review discusses some of them that may offer advantages over currently used agents and may warrant further investigation.

## 3. Results

### 3.1. Newer Antibiotics in the Quinolone/Fluoroquinolone Class

Quinolones and fluoroquinolones are bactericidal antibiotics that act on the enzymes DNA gyrase and topoisomerase IV, leading to the death of bacterial cells. Fluoroquinolones are among the “highest priority” and vitally important antibiotics for human medicine, according to the World Health Organization (WHO) [17].

In 2017–2019, some newer quinolones/fluoroquinolones, such as delafloxacin and lascufloxacin, were approved by the U.S. Food and Drug Administration (FDA) for treating specific infections, while other agents such as sitafloxacin and nemonoxacin obtained approval by other agencies, and other antibacterials, such as finafloxacin, which were previously approved, were later discontinued. Most of these antibiotics exhibited better pharmacological or antibacterial properties than the older agents [18,19]. However, it should be taken into consideration that tendon ruptures, peripheral neuropathy, hypoglycemia, retinal detachment, and an elevated risk of aortic aneurysm or dissection are the most serious side effects of fluoroquinolone therapy [20]. Therefore, patients who have aortic dilatation, are at risk of aortic aneurysm and dissection, or are geriatric should be administered these medications with caution. According to Rawla et al. [20], fluoroquinolone medication should only be given to such patients for a short time (7 to 14 days) and in the absence of other options for treatment.

#### 3.1.1. Delafloxacin

Delafloxacin is a potent anionic (or non-zwitterionic) fluoroquinolone for oral and intravenous administration [21,22]. In 2017 and 2019, the FDA approved the medication to treat acute bacterial skin/skin structure infections and community-acquired bacterial pneumonia. Increased antibacterial action against bacteria resistant to other fluoroquinolones and improved activity in acidic environments are provided by its three substituents [21,22]. Delafloxacin’s anionic nature makes it active in acidic settings such as inflammation, biofilms, abscesses, and stomach tissue, and its large molecular surface makes it more efficient against isolates resistant to previous fluoroquinolones [19]. Furthermore, according to the Phase 3 research by Horcajada et al. [23] comparing delafloxacin with moxifloxacin in patients with community-acquired bacterial pneumonia, only 0.5% of the 131 participants assessed in the delafloxacin group experienced CDI. Delafloxacin therefore carries a lower risk of CDI than previous fluoroquinolones.

Delafloxacin and levofloxacin activity against 96 anaerobic/microaerophilic clinical isolates was evaluated using E-tests and compared with the results of our earlier study [24]. Delafloxacin minimal inhibitory concentrations 90 (MICs_90_) were 0.75 mg/L for clostridia, 0.032 mg/L for other Gram-positive anaerobic rods, 0.38 mg/L for anaerobic cocci, and 0.5 mg/L for Gram-negative anaerobic rods. These values were significantly lower than those of levofloxacin. High delafloxacin MICs (≥4 mg/L) were found only in single *C. difficile*, *Clostridium clostridioforme*, *B. fragilis* and *Prevotella* isolates [24].

In a French study evaluating delafloxacin and levofloxacin activity on 231 *H. pylori* isolates using E-tests, delafloxacin ECOFF (epidemiological cutoff value) of ≤0.125 mg/L was recommended by Luzarraga et al. [25]. Delafloxacin MICs > 0.125 mg/L were found in approximately half (53.5%) of the isolates resistant to levofloxacin, whereas no delafloxacin resistance was found among levofloxacin susceptible isolates [25].

The primary cause of fluoroquinolone resistance in *H. pylori* strains is the substitution of an amino acid in the *gyrA* gene with the mutations D91G, D91N, N87K, or N87 [25]. Luzarraga et al. [25] found that the N87I mutation was frequently associated with resistance to both levofloxacin and delafloxacin, whereas resistance was less commonly linked to the point mutations D91N and N87K, which are common in European strains. The authors suggested that delafloxacin may show an advantage over levofloxacin in at least half of the strains resistant to levofloxacin [25].

We evaluated the effectiveness of delafloxacin and levofloxacin against 169 *H. pylori* isolates using E tests in our recent study [13]. Compared to levofloxacin resistance (30.8% at 1 mg/L according to EUCAST, v. 15.0 breakpoints in 2025), overall delafloxacin resistance was significantly lower (1.2% at 1 mg/L and 8.9% at 0.125 mg/L according to the proposed ECOFF). It is important to remember that certain *H. pylori* isolates that were resistant to levofloxacin also had resistance to delafloxacin at 0.125 mg/L (28.8% in our study compared to >50% in the French study) [23,25].

Therefore, the susceptibility of the isolates to delafloxacin should be examined if the more recent fluoroquinolone is used to treat a gastroduodenal infection linked to *H. pylori*. Additionally, Varshney et al. [26] used the AutoDock 4.2 tool to test FDA-approved antibiotics against the primary virulence factor of *H. pylori*, the cytotoxin-associated gene A (*cagA*) oncoprotein. They found that delafloxacin had a binding affinity to the oncoprotein, which can slow the development of the related diseases. Molecular docking simulation aims to identify inhibitors that can disrupt *H. pylori* pathogenic interactions with host cells, so that these agents can be further investigated, including in clinical trials.

Delafloxacin showed benefits over levofloxacin in terms of activity against anaerobes and potential utility for treating mixed anaerobic-aerobic infections, as well as *H. pylori* infection, according to the current data, and good potency in acidic environments. Nevertheless, more studies, including clinical trials, are needed to confirm its benefits.

#### 3.1.2. Finafloxacin

The new 8-cyano-fluoroquinolone antibiotic finafloxacin shows enhanced, acid-activated efficacy against *H. pylori* when compared to conventional fluoroquinolones [19]. Because of its unique chemical structure, it inhibits DNA replication in the stomach’s acidic environment (pH 5.0–6.0). Nevertheless, the drug may exhibit cross-resistance with other fluoroquinolone antibiotics. Despite showing promise in laboratory tests for *H. pylori* eradication, it is mostly approved as drops for otic usage rather than oral *H. pylori* treatment [19].

#### 3.1.3. Lascufloxacin

Lascufloxacin is a newer broad-spectrum 8-methoxy fluoroquinolone antibiotic with good tissue distribution and tissue absorption that can be administered orally or intravenously [27].

Yamagishi et al. [28] used the broth dilution method to study in vitro lascufloxacin activity against anaerobic isolates in comparison with that of three fluoroquinolones (levofloxacin, moxifloxacin, and garenoxacin) and four other comparator antibiotics. The MIC_90_ value of lascufloxacin was 2 mg/L versus 16 to >64 mg/L for the comparator fluoroquinolones against *C. difficile*, and 2 to 4 mg/L versus 16 to 64 mg/L against the Gram-positive anaerobic cocci *Finegoldia magna* and *Peptoniphilus asaccharolyticus*. However, lascufloxacin activity was moderate (MIC_90_, 4 mg/L) against Gram-negative anaerobes such as *B. fragilis* and *B. thetaiotaomicron*, although higher than that of levofloxacin (≥32 mg/L). The newer agent had MICs_90_ ≥ 4-fold lower against *Prevotella* spp. when compared to levofloxacin, whereas the difference between lascufloxacin and garenoxacin was less pronounced [28]. Nevertheless, lascufloxacin MICs_90_ against *B. fragilis* (4 mg/L) and *C. difficile* (2 mg/L) were 2-fold higher than those of metronidazole [28].

In a study on lascufloxacin activity in a mixed (*Streptococcus pneumoniae* and *Prevotella intermedia*) pneumonia model in neutropenic mice, Hagihara et al. [27] discovered that the more recent agent demonstrated strong activity against both facultatively anaerobic and obligate anaerobic species in both the single model and the mixed infection. Although lascufloxacin had an impact on the intestinal and salivary microbiota, Mukuda et al. [29] noted that its abundance returned in just one month.

#### 3.1.4. Nadifloxacin

The second-generation fluoroquinolone nadifloxacin was approved in Asia (Japan) and later (in 2000), the European Medicines Agency (EMA) also authorized its topical use. It was shown to suppress the DNA gyrase and activate T cells and keratinocytes and can be used as a 1% cream to treat *C. acnes* and other Gram-positive bacteria such as staphylococci in skin infections [18].

#### 3.1.5. Nemonoxacin

Nemonoxacin is a more recent C-8 methoxy quinolone that can be administered either intravenously or orally. Because it is non-fluorinated, it is less toxic. It demonstrated both in vitro and in vivo activity against common causative agents of community-acquired pneumonia, and carbapenem non-susceptible *Enterobacterales*, as well as some anaerobic and atypical pathogens [30,31]. The newer quinolone required changes in several bacterial genes and had a lower tendency to acquire resistance than the older agents in the class. Its low plasma protein binding rate of 16% implies that it penetrates infection sites well. Associated adverse events were often diarrhea, headache and dizziness.

The newer drug showed a lower MIC_90_ (8 mg/L) against *C. difficile* in both in vitro (by broth microdilution) and animal models of Lee et al. [32] when compared to ciprofloxacin (>32 mg/L) and moxifloxacin (16 mg/L), and was active against quinolone-resistant isolates, which increase the risk of CDI, although it had no effect on clostridial spore growth and germination. Furthermore, nemonoxacin inhibited *H. pylori* and was found to have up to 2-fold higher in vitro activity than levofloxacin [31].

However, more research is required to ascertain the advantages of nemonoxacin in treating *H. pylori* infection and CDI.

#### 3.1.6. Sitafloxacin

Sitafloxacin, a fourth-generation quinolone that targets both DNA gyrase and topoisomerase IV and has a chloride substitution at the eighth position of the quinolone ring, has stronger efficacy against anaerobic bacteria [33]. Only a few countries, such as Thailand and Japan, have authorized it for the treatment of genitourinary, respiratory, and oral infections as well as for *H. pylori* rescue regimens [33,34,35]. Additionally, the drug demonstrated efficacy against biofilm development, indicating that it may be utilized to treat implant-related infections [33].

Sitafloxacin outperformed levofloxacin against *H. pylori* in vitro, including for infections caused by *gyrA* mutant strains [36,37]. According to several investigations [34,38,39], susceptibility to sitafloxacin varied from 61 to 100.0%.

Sitafloxacin was frequently used in a VAS regimen, in conjunction with amoxicillin and vonoprazan, a strong oral potassium-competitive acid blocker, to treat *H. pylori* infection [40]. The triple VAS regimen was more successful in females and achieved eradication rates of over 90.0% [41,42]. However, because of potentially dangerous side effects, quinolone-based regimens are not recommended as first-line eradication treatment [12].

Data on some newer quinolones and fluoroquinolones are summarized in Table 1.

### 3.2. Newer Antibiotics in the Tetracycline Class

Third-generation tetracyclines include tigecycline, and the most recently (in 2018) FDA-approved eravacycline, omadacycline, and sarecycline [43,44].

The presence of efflux pumps, ribosomal protection proteins, or, less frequently, tetracycline-modifying/degrading enzymes can lead to acquired resistance in the tetracycline class antibiotics [45,46,47]. Newer agents such as eravacycline can overcome the usual resistance mechanisms encoded by ribosomal protective protein genes through a much stronger binding to the 30S ribosomal subunit [45]. Moreover, in Gram-negative bacteria, newer tetracycline antibiotics have structural modifications that render them low-affinity substrates for certain efflux pumps encoded by either *tet*(A) or *tet*(B) genes [46]. Nevertheless, resistance in the more recent agents may result from overexpression of additional efflux pumps or the presence of Tet(X) family of enzymes [46]. The newly emerged *tet*(X) genes can cause resistance to all tetracycline antibacterials. Anaerobes have adaptive resistance mechanisms as well. The Tet(X) enzymes were initially discovered in *B. fragilis*, but are currently found mainly in aerobic or facultative anaerobic bacteria such as *Enterobacterales* [47]. Differences were found in substrate selectivity and oxidation patterns between the anaerobes and aerobes or facultative anaerobes [47].

#### 3.2.1. Eravacycline

Eravacycline is a third-generation tetracycline (fluorocycline) antibiotic that is entirely synthetic. The drug stops bacteria from synthesizing proteins by reversibly attaching to the 30S ribosomal subunit. Both Gram-positive and Gram-negative facultative anaerobes are susceptible to the agent’s strong and wide-ranging in vitro and in vivo activity, and in 2018, FDA approval was granted for injectable eravacycline treatment of complicated intraabdominal infections [48].

Two changes set the newer agent apart from tigecycline: a fluorine atom at position C-7 and a chain at position C-9 of the tetracycline core [49]. Gram-negative (*Enterobacterales*, *Acinetobacter baumannii*) and Gram-positive (staphylococci, enterococci, and pneumococci) bacteria were among the aerobes and facultative anaerobes that eravacycline demonstrated activity against.

Eravacycline was more effective against anaerobic bacteria in vitro (frequently with ≥4 to ≥8-fold lower MICs) than the majority of comparator antibiotics [50]. The fact that eravacycline MICs_90_ were 2–16 times lower than those of tigecycline against *Bacteroides/Parabacteroides* spp. is crucial because BFG species are frequently MDR anaerobic isolates from infections. In research conducted in Taiwan, the United States, and Canada [51,52,53], eravacycline MICs_90_ against *Prevotella* and *Fusobacterium* spp. were at least eight times lower than those of comparator antibiotics [52,54]. For these studies, the agar dilution method was used by Goldstein et al. [51] and Snydman et al. [52]; the broth microdilution method was applied by Tsai et al. [53]; and the E-test was used by Wolf and Stingu [54]. Although different methods were used to test susceptibility, the results are indicative; furthermore, Wolf and Stingu [54] found a high degree of agreement (>90%) between the results of the agar dilution method (the gold standard) and the E-test for two antibiotics under comparison.

Bassères et al. [55], using the broth microdilution method, found that eravacycline had lower MIC_90_ (0.016 mg/L) than fidaxomicin (0.063 mg/L), metronidazole (1.0 mg/L), and vancomycin (4.0 mg/L) against *C. difficile*, and the newer agent also had bactericidal activity against the highly epidemic and hypervirulent ribotype RT027. Moreover, eravacycline activity was not influenced by efflux pumps and genes encoding tetracycline resistance such as *tetQ* in *B. fragilis* isolates and *tetM* and *tetW* in *C. difficile*, and did not induce CDI [52,55,56].

The fluorocycline was also potent in vitro against the anaerobic cocci, *Porphyromonas* and *Cutibacterium* spp., as well as many rarely isolated anaerobes such as *Capnocytophaga*, *Lancefieldella*, *Actinotignum*, *Eggerthia*, and *Fannyhessae* spp. [52,54].

The findings demonstrate potent in vitro efficacy of eravacycline against anaerobic bacteria, but it is necessary to conduct similar studies more widely and in more countries around the world.

#### 3.2.2. Omadacycline

Like eravacycline, omadacycline is a newer third-generation tetracycline. It is a semisynthetic aminomethylcycline, FDA-approved for treating community-acquired bacterial pneumonia and acute bacterial skin and skin structure infections [43,44]. Omadacycline can be taken orally or intravenously. On the other hand, food can lower oral medication concentrations by more than 40%. Omadacycline has activity comparable to that of eravacycline, but not as potent in vitro against Gram-negative bacteria.

The agent exhibited activity against Gram-positive anaerobes such as *C. difficile* (MICs, 0.25 to 8 mg/L and MIC_50_, 0.5 mg/L), *C. perfringens*, and Gram-positive anaerobic cocci (MICs, 0.06 to 2 mg/L), whereas against Gram-negative anaerobes, such as some *Bacteroides* and *Prevotella* spp., MIC ranges were from 0.06 to as high as >16 mg/L [43,44]. The agar dilution method was used for *C. difficile* and *Prevotella* spp., and the broth microdilution method was used for *Bacteroides* spp. [43].

The effectiveness of eravacycline, omadacycline, and tetracycline against 201 Chinese *H. pylori* isolates was compared by Yang et al. [57], using the agar dilution method. With MIC_50_ and MIC_90_ values of 0.125 and 0.25 mg/L for omadacycline and 0.063 and 0.125 mg/L for eravacycline, respectively, compared to 0.25 and 1 mg/L for tetracycline, the more recent antibiotics were more effective than tetracycline against *H. pylori* isolates. Therefore, both newer tetracycline-class agents can be further investigated for the management of *H. pylori* infection, particularly in countries with high rates of tetracycline resistance.

#### 3.2.3. Sarecycline

With a global incidence of 9.4 to 20.5%, acne vulgaris is a common chronic skin condition that affects between 28% and 85% of teenagers and young adults worldwide [58,59,60]. Moreover, some authors have reported an increase in adult acne [60] and a high (47%) pooled prevalence of scars in acneic patients [61]. Treatment of the disorder involves non-antibiotic agents such as benzoyl peroxide, retinoids, and isotretinoin, as well as laser and photodynamic therapy, and antibiotics such as macrolides and tetracyclines, especially when topical therapy fails [62].

Sarecycline is a semisynthetic and narrow-spectrum oral (once daily) derivative of tetracycline, FDA-approved in 2018, and intended for treating inflammatory non-nodular lesions of moderate to severe acne vulgaris in patients aged ≥9 years, with beneficial effects that can manifest as early as three weeks after sarecycline administration [58]. Lomakin et al. [59] observed that sarecycline bound to two ribosome sites, the A-site of the 30S subunit and to a nascent peptide exit tunnel (NPET) site on the 50S subunit, thus preventing both the start of peptide elongation and the translocation.

Sarecycline (1.5 mg/kg/day) significantly reduced inflammatory lesions in the Phase 3 studies, and while side effects such as gastrointestinal disturbance (<5% of patients), nausea (1.2%), and vaginal candidiasis (1.2%) were reported, the drug can infrequently (0–<1%) cause phototoxicity, pseudotumor cerebri, and lupus [58,63]. Due to its narrow spectrum, sarecycline has no activity against Gram-negative bacteria, thus preserving their equilibrium in the intestinal microbiota and minimizing the development of antibiotic resistance.

In brief, sarecycline is a successful antibiotic for treating acne despite being more costly than other tetracyclines because it combines a number of advantageous characteristics, including high activity against *C. acnes*, low rates of antibiotic resistance, uncommon adverse effects, and minimal disruption to the gut microbiota.

In short, data on the newer tetracyclines are presented in Table 2.

### 3.3. Activity of Other Newer Antibiotics Against Anaerobes and Helicobacter pylori

#### 3.3.1. Amixicile

Overgrowth of anaerobic bacteria can cause periodontal disorders, including periodontitis, representing a dysbiotic bacterial community, and affecting 1/5 to ½ of the global human population [64]. Periodontitis increases the risk of tooth loss, alveolar bone resorption, and gingival inflammation [65]. *Porphyromonas gingivalis*, a Gram-negative anaerobic species, is crucial to the development of periodontal biofilm and inflammation [65]. Patients with periodontitis were also more likely to have other oral anaerobes like *Treponema denticola*, *Tannerella forsythia*, and *Parvimonas micra* [66], and *Prevotella intermedia* and *Fusobacterium nucleatum* have also been reported [67]. Antibiotics such as clindamycin, amoxicillin and tetracycline, can be used as an adjunct for treatment of periodontitis [68].

By blocking the activity of pyruvate ferredoxin oxidoreductase (PFOR), an essential enzyme that drives pyruvate’s oxidative decarboxylation, amixicile, a more recent and most probably non-toxic agent with no negative side effects, selectively targets specific oral anaerobes [69,70]. Humans and aerobes/facultative anaerobes do not contain PFOR, and the agent has small effect on gut microbiota. Additional benefits of amixicile include its ability to target anaerobic bacteria in biofilms and, in contrast to broad-spectrum drugs, its inability to promote antibiotic resistance [67].

Animal studies showed the activity of amixicile on *C. difficile*, *Bacteroides* spp., and *H. pylori*; however, the cross-resistance between the agent and metronidazole limits amixicile use for *H. pylori* infections [71].

An ex vivo microbiome was produced by Gui et al. [69] from the gingival pockets of subjects with severe periodontitis. The authors investigated the efficacy of amixicile by inducing a particular knockdown suppression of anaerobic bacteria in the microbiome. They found that amixicile selectively diminished the abundance of anaerobic periodontal pathogens such as *Prevotella*, *Porphyromonas*, *Slackia*, and other species [69]. As a result, the drug may be utilized to prevent periodontal disease and stop the proliferation of these bacteria in people with periodontitis [69]. Amixicile has been proposed by Hutcherson et al. [67] as a potential treatment for periodontitis. However, additional studies, mainly clinical, are needed for this purpose.

#### 3.3.2. Beta-Lactam in Combinations

Beta-lactams are a frequently used group of antibiotics. They suppress bacterial cell wall biosynthesis and peptidoglycan cross-linking [72]. At present, there are many newer beta-lactam/beta-lactamase inhibitor antibiotics, such as aztreonam/avibactam, cefepime/enmetazobactam, meropenem/vaborbactam, and imipenem/relebactam, with activity against MDR aerobic and facultative anaerobic bacteria [72]. Snydman and McDermott [73] evaluated the in vitro activity of aztreonam/avibactam against numerous (341) isolates of anaerobes in 2016–2020. The authors found that avibactam added to aztreonam did not increase the activity of the combination against anaerobes and did not influence *cfiA* gene-produced metalloenzyme of BFG [73]. Similarly, Darlow et al. [74] have reported a low activity of cefepime/enmetazobactam against anaerobes. Meropenem exhibits antimicrobial activity against some anaerobic bacteria, but Patel et al. [75] found that the anaerobic activity of meropenem–vaborbactam was comparable to that of meropenem alone. Another newer agent is imipenem/relebactam. Like imipenem, imipenem/relebactam was highly effective against anaerobic bacteria with a low resistance rate of roughly 0.7%; however, the imipenem activity against anaerobes did not increase by the addition of relebactam [76].

In short, newer beta-lactam combinations with beta-lactamase inhibitors have not yet been shown to be more effective against anaerobic bacteria than the antibiotic in the combination when used alone.

#### 3.3.3. Cadazolid

As the first antibacterial agent in the quinoxolidinone (fluoroquinolone-oxazolidinone) antibiotic class, cadazolid has activity against *C. difficile* by inhibiting the bacterial DNA and protein synthesis [77,78].

Clinical cure rates of cadazolid (250 mg/dose, b.i.d.) compared to vancomycin (in noninferiority Phase 3 clinical trials on >1260 patients from 27 countries) showed no superiority of cadazolid over vancomycin against *C. difficile*; therefore, the use of the quinoxolidinone agent was neither advised nor subsequently developed for clinical purposes [77]. Nevertheless, the CDI recurrence rate was much lower (8.1%) in the cadazolid-treated patients compared with the vancomycin-treated group (17.3%) [77], and the low impact of the newer antibiotic on the gut microbiota was emphasized by Quan et al. [77] and Rueedi et al. [78].

#### 3.3.4. CRS3123

CRS3123 is a narrow-spectrum, 1-benzopyran organic aromatic that may be helpful in the management of CDI [79]. The drug targets methionyl-tRNA synthetase type 1 (methionyl-tRNA ligase type 1) of Gram-positive bacteria such as staphylococci, streptococci and enterococci, and also the anaerobic species *C. difficile*, including the hypervirulent RT027 (MICs, 0.5 to 1 mg/L, using the agar dilution method), [79,80]. The majority of intestinal anaerobes and Gram-negative bacteria depend on another enzyme, against which CRS3123 is inactive. CRS3123 at 1 mg/L can suppress the production of *C. difficile* toxins and sporulation by displacement of the methionine from the target, hence preventing tRNA charging and protein synthesis [80].

The efficacy and safety profile of oral CRS3123 (200 and 400 mg b.i.d.) compared to oral vancomycin (125 mg q.i.d.) in people with CDI (initial infection or first recurrence) were the main focus of Louie et al. [80] in Phase 2, a double-blind, randomized, and multicenter study. Thirteen patients treated with 200 mg of CRS3123, fourteen patients treated with 400 mg of CRS3123, and thirteen subjects treated with 125 mg of vancomycin had SCRs that were higher (92–93%) in the CRS3123 group than in the vancomycin group (77%). The recurrence rates were lower (0–7% by day 40 and 0–14% by day 70) among the patients treated with CRS3123 than in the vancomycin-treated group (23%). Gastrointestinal pain was the most common mild or moderate adverse event associated with the newer agent [80].

The results indicate that CRS3123 is a promising candidate for optimizing CDI therapy.

#### 3.3.5. Gepotidacin

Gepotidacin represents a first-in-class triazaacenaphthylene antibiotic with bactericidal activity, suppressing bacterial DNA replication by distinct binding-site inhibition of both DNA gyrase (GyrA enzyme subunit) and topoisomerase IV (ParC enzyme subunit) [81]. Gepotidacin was found to be active and well tolerated for treating uncomplicated urinary tract infections, and according to the Phase 3 multicenter and non-inferiority trial, it was non-inferior to ceftriaxone + azithromycin for treating urogenital infections caused by *Neisseria gonorrhoeae* [82].

Hackel et al. [81] tested gepotidacin activity against 558 Gram-negative and Gram-positive anaerobes using the agar dilution and broth microdilution methods. By the agar dilution method, gepotidacin MIC_90_ (4 mg/L) was 2 to 8-fold higher than those of metronidazole and imipenem, but ≥2-fold lower than those of clindamycin and piperacillin–tazobactam. The MIC_90_ was 2-fold lower (2 mg/L) by broth microdilution than by agar dilution (4 mg/L) when tested against BFG isolates. At 1 and 2 mg/L, the agent inhibited 79.0% and nearly 89%, respectively, of all Gram-negative anaerobic isolates [81]. Gepotidacin MIC_90_ against all Gram-positive anaerobic isolates (2 mg/L) was 4-fold higher than that of metronidazole, but 4-fold lower than those of clindamycin and imipenem, and 8-fold lower than that of piperacillin–tazobactam. At 1 and 2 mg/L, the agent inhibited about 71% and 90% of all Gram-positive anaerobic isolates [81].

Gepotidacin showed in vitro activity against clinically significant anaerobic bacteria, although not stronger than that of metronidazole. Additional investigation is needed to thoroughly evaluate the full activity of the newer agent.

#### 3.3.6. Ibezapolstat

A recent study revealed the potential benefits of the newer antibiotic, a guanine analog and the first-in-class bacterial protein PolC (DNA polymerase IIIC) inhibitor, ibezapolstat. PolC bacterial protein is present only in Gram-positive bacteria such as *C. difficile* [83]. Ibezapolstat has a narrow spectrum of activity. In an open-label, (Phase 2a) study, it showed a narrower-than-expected spectrum of activity, leading to preservation of the bacteria (*Lachnospiraceae* and *Oscillospiraceae*) participating in the bile acid metabolism, and provided a sustained clinical response in all 10 participants in the trial. The agent had favorable pharmacokinetics, good tolerability and no clinically significant adverse effects [83].

In a Phase 2b clinical trial (a randomized and double-blind study on adult patients with CDI in 15 US centers), orally given ibezapolstat (three capsules of 150 mg, b.i.d. for 10 days) or placebo capsules (b.i.d.) were compared to orally given vancomycin (one capsule of 125 mg, q.i.d, for 10 days) or placebo capsules (b.i.d.) [83]. Comparable initial clinical cure was observed in 94% (15/16 patients) in the ibezapolstat group and in all 14 patients in the vancomycin group. However, on day 28 following the conclusion of treatment, none of the patients in the ibezapolstat group experienced a recurrence, compared to 14% in the vancomycin group. Notably, 94% of patients in the ibezapolstat group had SCRs, compared to 86% in the vancomycin group (*p* = 0.59). In both groups, none of the patients experienced severe side effects or stopped taking the newer agent [83].

In short, in adult patients with CDI, ibezapolstat and vancomycin demonstrated comparable effectiveness, good SCR, comparable mean time for diarrhea resolution, and good tolerability. Phase 3 trials are necessary to further assess the benefits of ibezapolstat, which showed high concentration in feces, maintenance of the microbiota and the equilibrium of bile acids, as well as a minimal risk of recurrence of the infection.

#### 3.3.7. Lefamulin

In 2019 and 2020, the FDA and EMA approved lefamulin, a novel tricyclic diterpenoid pleuromutilin antibiotic, for use in oral and intravenous formulations to treat adult patients with community-acquired bacterial pneumonia [84,85]. The drug exhibits a unique method of action that prevents protein synthesis by binding to the peptidyl transferase core of the 50S bacterial ribosome subunit and blocking the binding of tRNA for peptide transfer via a so-called “induced-fit mechanism” [84]. Lefamulin is effective against methicillin-resistant *Staphylococcus aureus* and vancomycin-resistant enterococci as well as other bacteria such *S. pneumoniae*, *Haemophilus influenzae*, *Mycoplasma*, *Legionella*, and *Chlamydophila* spp. An intravenous dose of 150 mg b.i.d. or an oral dose of 600 mg b.i.d. of the pleuromutilin antibiotic is well tolerated. Reported adverse effects have been gastrointestinal effects; risk for development of CDI, risk of cardiac arrhythmia, and fetal damage in pregnancy [84,85].

Lefamulin demonstrated activity against some anaerobic species, such as *Clostridium perfringens*, *C. acnes*, *Prevotella* and *Fusobacterium* spp., as well as Gram-positive anaerobic cocci; however, no activity was demonstrated against *C. difficile* or *B. fragilis* [86]. This may limit the use of the newer antibiotic for anaerobic infections.

#### 3.3.8. Ridinilazole

Ridinilazole is an antibiotic under investigation, a narrow-spectrum bis-benzimidazole agent with bactericidal activity against *C. difficile*, suppressing its cell division [87]. The agent binds itself to *C. difficile* AATTT-rich sequences in the DNA minor groove, impacting cell septum development and, most likely, ATP production [87].

In a Phase 2 trial, CDI adult patients received either oral ridinilazole (200 mg b.i.d.) or oral vancomycin (125 mg q.i.d.) for 10 days. By a modified intention-to-treat analysis, significantly more (66.7%) patients treated with ridinilazole had SCRs versus 42.4% of those treated with vancomycin [88]. Unlike vancomycin, ridinilazole preserved the profile of intestinal bile acids, which can affect both *C. difficile* germination and growth [89].

Phase 3 trials were performed on 759 CDI patients, treated with either oral ridinilazole or oral vancomycin (as above) for 10 days, and showed a significantly lower (8.1%) recurrence rate in the ridinilazole group versus vancomycin group (17.3%); however, ridinilazole did not outperform vancomycin in the SCR [88,90]. In the meta-analysis of Bednárik et al. [10], ridinilazole and fidaxomicin were found to be the best antibiotics for the prevention of relapsing CDIs.

Briefly, ridinilazole did not perform better than vancomycin in terms of SCR, but a subgroup analysis revealed that the bis-benzimidazole drug conserved the intestinal flora and decreased the risk of recurrent infection. Thus, some potential biases should be addressed [90], and further research is required before its clinical use can be recommended.

#### 3.3.9. Rifasutenizol (TNP-2198)

Finding novel antibacterial drugs is one way to combat antibiotic resistance; another is to develop hybrid compounds that make use of multiple mechanisms [91]. One such hybrid antibiotic is the investigational agent rifasutenizol (TNP-2198), which displays two mechanisms of action, representing a conjugation of both rifamycin and nitroimidazole pharmacophores. Rifasutenizol suppresses bacterial DNA-directed RNA polymerase and shows a greater activity than a 1:1 molar mixture of the parent antibiotics [92].

The agent showed activity against clinically important microaerophiles such as the widespread *Helicobacter pylori* and anaerobic pathogens, such as *C. difficile* and *Gardnerella vaginalis*, the bacteria associated with bacterial vaginosis [92]. In a hamster model using a strain of *C. difficile* UNT103-1, rifasutenizol (45 mg/kg) treated animals showed a 100% survival rate on day 21 (the end of the trial) versus 80–100% for those treated with vancomycin (20 mg/kg) [92]. Rifasutenizol exhibited faster bactericidal activity and less-frequently-selected antibiotic-resistant mutants than the comparator antibiotics [93]. The agent was evaluated in three trials and exhibited favorable safety, tolerability and pharmacokinetic profiles, and in another trial, it was also active against *H. pylori* [93].

Comparing *H. pylori* eradication rates by RTT (oral capsules of rifasutenizol 400 mg + amoxicillin 1 g + rabeprazole tablets 20 mg) to BCTT (oral tablets of bismuth potassium citrate 240 mg + clarithromycin 500 mg + oral amoxicillin capsules 1 g + rabeprazole tablets 20 mg), all b.i.d. for 14 days, RTT showed non-inferiority (92.0%) to the BCTT (87.9%) [94].

However, it should be taken into account that rifasutenizol is still an antibiotic under investigation, and more studies are needed to reveal its therapeutic benefits.

#### 3.3.10. Zoliflodacin

Zoliflodacin, a more recent spiropyrimidinetrione antibacterial agent that binds to the GyrB subunit of the bacterial DNA gyrase, exhibited activity against *Neisseria gonorrhoeae* as a potential oral agent for uncomplicated gonorrhea, *Mycoplasma genitalium*, and *H. pylori* [95,96]. Advantages of the newer antibiotic are its anti-biofilm properties and synergism with other antibiotics, such as ampicillin/sulbactam and carbapenems, as demonstrated for *Acinetobacter baumannii* [95,96].

Liu et al. [96] studied zoliflodacin activity in vitro against 123 MDR isolates of *H. pylori*. The authors detected low MICs of the agent with MIC_50_ and MIC_90_ of 0.125 and 0.25 mg/L, respectively. In order to find GyrB mutations associated with zoliflodacin resistance, the authors examined more than 2200 *H. pylori* whole-genome sequences. Since mainly the GyrB subunit of the DNA gyrase is targeted by the agent, resistance can be conferred through either Asp424Asn or Lys445Asn alterations, but they were found to be uncommon among the enormous quantity (2262) of *H. pylori* sequences assessed by whole-genome sequencing [61].

Therefore, zoliflodacin can become an alternative antibiotic in the regimens for *H. pylori* eradication, provided that its activity is confirmed in clinical trials.

The results for the aforementioned antibiotics are presented in Table 3.

## 4. Discussion

Most newer antibiotics are not affected by the presence of antibiotic resistance genes and efflux pumps, unlike older agents in the same class. Many of them also have additional advantages and some disadvantages.

The FDA or EMA has approved many of the more recent antibiotics, including delafloxacin, lascufloxacin, lefamulin, eravacycline, and omadacycline, for the treatment of MDR bacterial infections caused by aerobes or facultative anaerobes [97]. However, some studies have indicated that these antibiotics may also be used to treat anaerobic and microaerophilic infections, which warrants further investigation. Only a few newer antibacterials, such as sarecycline, have received FDA approval for the treatment of anaerobic (*C. acnes*) skin infections [97]. Other antibiotics, such as nadifloxacin, are approved in several Asian and some European countries, and in Japan as a topical cream for the treatment of acne vulgaris, but have not received FDA approval [18].

Rifasutenizol, gepotidacin, amixicile, and CRS3123 are examples of newer antibacterials that are still undergoing research.

An important characteristic of newer antibiotics is to show advantages over the agents of the same class that are currently in use. In addition to the higher activity in vitro and resistance barriers to the genetic factors conferring antibiotic non-susceptibility, anti-biofilm activity is another advantage of some newer antibiotics. Biofilm formation is common in chronic infections and is associated with increased resistance of the bacteria to antibiotics, immune defense, and environmental factors compared to planktonic bacteria [98].

Some newer agents show other beneficial properties, such as activity under acid conditions and low or no impact on the human microbiome [19,21,22].

On the other hand, certain adverse effects (see below) of newer antibiotics and their significantly higher cost, as well as the lack of clinical studies on some agents, are factors that must also be considered.

Below are presented examples regarding the advantages and disadvantages of some newer antibiotics with activity against anaerobic and microaerophilic bacteria compared to other antibacterials in the same class or to other frequently used therapeutic agents.

### 4.1. Comparison Between Newer and Older Antibiotics

In comparison to levofloxacin, the newer fluoroquinolone delafloxacin was more potent in vitro against many anaerobic species and *H. pylori* [13,19,24,25,26]. Another recent fluoroquinolone, lascufloxacin, was more active against *C. difficile*, *Prevotella* spp., and anaerobic cocci, than the comparator fluoroquinolones such as levofloxacin [27,28]. However, lascufloxacin had lower activity (MICs_90_) against *B. fragilis* and *C. difficile* than metronidazole [28]. The fluorocycline, eravacycline, exhibited better activity in vitro against anaerobes such as *C. difficile* compared to metronidazole and vancomycin, against BFG compared to tigecycline and against many other anaerobic species compared to comparator antibiotics [51,52,53,54]. Moreover, both delafloxacin and eravacycline did not pose a risk for CDI, which is important for patients who have experienced recurrent infections [23,99]. In the study of Zhang et al. [100], in the USA during the period 2008–2020, the highest risk of developing CDI was linked to the use of doxycycline, clindamycin, and currently used fluoroquinolones. However, the authors encompassed moxifloxacin, levofloxacin, and ciprofloxacin in a single antibiotic group [100].

Against *H. pylori*, both eravacycline and omadacycline had better potency in vitro than tetracycline as a comparator [57]. Delafloxacin can outperform previous fluoroquinolones used against *H. pylori* infection in several ways, including not only increased potency in vitro but also enhanced activity in acidic environments such as the gastric mucosa and activity against biofilms [13,21,22,25]. Sitafloxacin was also more active in vitro than levofloxacin against *H. pylori* [36,37]. In addition, the agent has anti-biofilm properties [33]. Anti-biofilm activity has been reported for amixicile against anaerobic biofilms and for zoliflodacin [67,95,96]. However, it is desirable to investigate the anti-biofilm activity of most of the newer antibiotic agents.

Against *C. difficile*, both cadazolid and ridinilazole were superior to the currently used antibiotics in terms of CDI recurrence rates and the effect on the gut microbiota [77,78,88,90]. Similarly, both CRS3123 and ibezapolstat outperformed vancomycin in patients with CDI in terms of recurrence rates [80,83]. Nemonoxacin showed better in vitro activity than ciprofloxacin and moxifloxacin against *C. difficile*, including quinolone-resistant isolates, as well as against *H. pylori* compared to levofloxacin [31,32].

An interesting approach is the development of hybrid antibiotics such as rifasutenizol (TNP-2198), which joins two (rifamycin and nitroimidazole) pharmacophores in one molecular entity and showed activity against both rifamycin- and nitroimidazole-resistant isolates with resistance to one or both agents [92].

### 4.2. Cross Resistance and Resistance Barriers

Cross-resistance with other antibiotics used for a given infection can reduce the therapeutic usefulness of newer antibacterials. An advantage of the newer antibiotic finafloxacin is its increased activity in an acidic pH environment; however, it also has the disadvantage of cross-resistance with other fluoroquinolones [19]. Amixicile can exhibit cross-resistance with nitroimidazoles, which is high in *H. pylori* [71].

Both delafloxacin and sitafloxacin were active against *H. pylori* strains resistant to older fluoroquinolones [25,36,37]. Sitafloxacin was inhibitory to *gyrA* mutant isolates [36,37], and delafloxacin showed activity in vitro against about half of levofloxacin-resistant isolates [25]. Delafloxacin activity was not affected by the amino acid exchanges D91G and D91Y, which were frequently linked to fluoroquinolone resistance in European *H. pylori* isolates [25]. However, the presence of *gyrA* mutations such as N87I that led to resistance to both delafloxacin and levofloxacin [25] shows the need to test for antibiotic susceptibility of the strains to delafloxacin prior to its use for the eradication of *H. pylori*.

Eravacycline was unaffected by efflux pumps or some genes such as *tetM*, *tetQ*, and *tetW*, that encode tetracycline resistance in *B. fragilis* or *C. difficile* [50,52,54,55,56].

### 4.3. Side Effects

Some newer antibiotics, such as rifasutenizol, have been found to be both safe and well-tolerated in several clinical trials [93], while others may cause side effects and/or have certain restrictions for use.

Importantly, eravacycline shares the same adverse effects as antibiotics in the tetracycline class and should not be used to treat children younger than 8 years or women in the second/third trimester of pregnancy because it can discolor teeth and delay the growth of bones [48]. The side effects of tetracycline-class antibiotics are often gastrointestinal disturbances. They were up to 5-fold more frequently observed for minocycline, tigecycline, and omadacycline than for eravacycline [48].

Lefamulin is considered well-tolerated, but it may carry a risk of developing CDI or cardiac arrhythmia, as well as of fetal harm [84,85].

As mentioned above in Section 3.1, the use of fluoroquinolones can lead to serious side effects, some of which are severe and irreversible [20]. In the review article of Rusu et al. [101], the safety profile of the most recent fluoroquinolones, including delafloxacin, lascufloxacin, nemonoxacin, and sitafloxacin, has been discussed. In comparison to older fluoroquinolones, no serious adverse reactions, such as acute renal failure, tendinitis, tendon rupture, retinal detachment, and severe aortic aneurysms/aortic dissection, have been reported for the newer agents [101]. Furthermore, unlike many currently used fluoroquinolones, delafloxacin use is not a risk factor for hypervirulent CDI. Delafloxacin was not linked to phototoxicity in clinical trials. There have occasionally been reports of delafloxacin-related peripheral neuropathy, and nemonoxacin has been linked to peripheral neuropathy symptoms or QT prolongation at high doses [101].

An important adverse effect associated with the use of fluoroquinolones is dysglycemia. For example, hypoglycemia associated with levofloxacin and moxifloxacin can be severe and life-threatening. Symptomatic hypoglycemia may also be due to the use of delafloxacin in single cases, but has not yet been reported for nemonoxacin and sitafloxacin [101]. However, it is advised that the most recent fluoroquinolones be used carefully and only in cases of serious, life-threatening infections for which there are no other available treatment options [101].

### 4.4. Impact on the Intestinal Microbiota

In comparison with the broad-spectrum antibiotics such as fluoroquinolones, including the newer agents, and tetracyclines, including eravacycline, narrow-spectrum antibiotics such as amixicile, CRS3123, ibezapolstat, ridinilazole, and sarecycline affect to a lesser extent the gut microbiota and do not pose a risk of an increase in antibiotic resistance [58,67,69,70,79,83,87]. If clinicians have the option to choose, the newer narrow-spectrum antibiotics should be preferred over those with a broad spectrum of activity.

### 4.5. Price of Newer Antibiotics

The higher cost of new antibiotics compared to commonly used ones is a major barrier to their introduction and use in many countries. Mitra-Majumdar et al. [102] have reported the cost factors (cost ratios, higher acquisition prices compared to older generic drugs) of the newer antibiotics to be 1.47 for eravacycline compared to ertapenem, both for parenteral treatment course, ≥3.83 for omadacycline intravenously compared to linezolid intravenously, and >33 for oral lefamulin compared to oral moxifloxacin, whereas oral delafloxacin was less expensive than intravenous vancomycin with a cost factor of 0.48.

The cost of seeking, evaluating, and conducting in vivo studies of newer antibiotics is also significant, but although some initial or small studies may not reveal all potential limitations of newer agents, they can contribute to life-saving therapy for certain patients. Subsequent clinical trials and post-marketing surveillance can reveal the full clinical usefulness of the newer antibacterials.

## 5. Conclusions and Future Directions

In conclusion, there are fewer clinical studies on the activity of newer antibiotics against anaerobes and microaerophiles compared to aerobic bacteria. Most studies have been conducted on *C. difficile*. They demonstrate the advantages of certain newer antibiotics over older agents, such as:

- the advantage of delafloxacin, which shows minimal risk of inducing CDI, unlike older fluoroquinolones, which are a risk factor for infection by hypervirulent strains,
- the benefit of sarecycline as a narrow-spectrum antibiotic effective for treating acne vulgaris, as well as
- the achievement of lower recurrence rates of CDI with CRS3123, ridinilazole, cadazolid, and ibezapolstat than with vancomycin in patients with CDI, although cadazolid and ridinilazole did not meet the requirements for noninferiority compared to vancomycin.

With regard to *H. pylori*, clinical trials revealed that:

- sitafloxacin showed a very high eradication rate when combined with amoxicillin and vonoprazan (VAS regimen); however, regrettably, neither sitafloxacin nor vonoprazan has yet been approved by the EMA and neither is currently used in Europe.
- Another newer antibiotic, rifasutenizol, exhibited similar efficacy in eradicating *H. pylori* infection in a triple regimen with amoxicillin and rabeprazole compared to a quadruple regimen with bismuth, clarithromycin, amoxicillin and rabeprazole. Nevertheless, rifasutenizol is still an investigational new antibiotic.

The above-mentioned antibiotics should be studied more extensively to improve treatment or as a therapeutic alternative for *C. difficile* or *H. pylori* infections, for which treatment optimization is needed.

Both antibiotic preservation and newer antibiotic research and development must be balanced. Despite the continuing rise in antibiotic resistance and the growing significance of effective antimicrobial drugs, many countries currently fail to shape the market [103].

The use of newer antibiotics, such as lefamulin, delafloxacin, eravacycline, and omadacycline is problematic even in Europe. Numerous scientific, economic, and regulatory factors contribute to that, including the lack of researchers (there were only about 500 working on antibiotic development in 2017), the difficulty of getting regulatory approval for antibacterials at the in vitro stage, financial risk factors, the cost of the newer agents, and other reasons [103,104].

Effective antibiotics are difficult to obtain for both governments and individual patients, mostly but not only in developing countries [103], especially newer agents that could be useful in cases when treatment with commonly used drugs fails.

According to Berman et al. [103], more emphasis should be put on ensuring that more countries have access to newer antibacterials, both present and future. It is also desirable to more actively support research on antibiotics currently under investigation. In order to achieve this goal, the authors recommended creating additional regional working groups to conduct the necessary studies, to determine the financial scope based on the expected turnover, to thoroughly examine supplier and market issues, and to collaborate with expert partners [103]. Anderson et al. [104] suggested holding in-depth discussions with academic, industrial, governmental, and medical representatives, as well as paying for subscriptions, and other strategies. Additionally, more efforts are needed to establish networks for clinical trials and scientific expertise, as well as to organize larger-scale in vitro and clinical studies necessary for the clinical development of newer agents [104].

Important steps have already been taken in some European countries. In France, there is a pricing system intended to prevent the majority of medications from being reimbursed at rates lower than those in four major reference markets in Europe, and since 2021, this has also applied to antibiotics approved according to non-inferiority clinical trial results [104].

New approaches, such as nanomaterials [105], antimicrobial peptides, efflux pump inhibitors, phage therapy, human monoclonal antibodies, and clustered regularly interspaced short palindromic repeats (CRISPR)-Cas, a revolutionary gene-editing tool [106], should be further evaluated to contribute to the treatment of infections caused by antibiotic resistant and MDR microorganisms, including anaerobic and microaerophilic bacteria.

Although there are many challenges, there are also many opportunities to promote research, development, and the potential use of newer antibiotics that have proven to be superior to those currently in use, so that many newer agents become available in various countries and regions to the patients who are most in need of them.

## Figures and Tables

**Table 1:** Activity of some newer antibiotics of quinolone/fluoroquinolone class against anaerobes and *Helicobacter pylori*.

Antibiotics, Activity Against	*C. difficile*	*Bacteroides* spp.	Other Anaerobes	*H. pylori*	References
**Delafloxacin** (a 4th generation anionic fluoroquinolone)	Better in vitro activity compared to LVX. Enhanced activity in acidic environments. Does not induce CDI.	Better in vitro activity compared to LVX. Enhanced activity in acidic environments.	Better in vitro activity compared to LVX. Enhanced activity in acidic environments.	Better in vitro activity compared to LVX. Binding affinity to *H. pylori* CagA. Enhanced activity in acidic environments.	[13,19,24,25,26]
**Finafloxacin** (a 4th generation, 8-cyano-fluoroquinolone antibiotic)				Acid-activated activity under acidic environment. May display cross resistance with older quinolones. So far approved for otitis externa.	[19]
**Lascufloxacin** (a newer 8-methoxy fluoroquinolone)	LSFX MIC_90_ was ≥8-fold lower than that of 3 comparator FQs, but higher than that of MTZ.	Moderate activity (MIC_90_, 4 mg/L), but lower than that of MTZ.	MICs_90_ ≥ 4-fold lower against three *Prevotella* spp., compared with LVX. Activity against *P. intermedia* in a mouse model.		[27,28]
**Nadifloxacin** (a 2nd generation fluoroquinolone)			A topical agent for treating *C. acnes* and staphylococcal skin infections.		[18]
**Nemonoxacin** (a newer, non-fluorinated C-8 methoxy quinolone)	Lower MIC_90_ than CIP and MFX. No effect on spore germination.			A twofold increased activity in vitro compared to LVX.	[30,31,32]
**Sitafloxacin** (a 4th generation fluoroquinolone antibiotic)			Activity against anaerobic bacteria. Anti-biofilm properties.	More potent than LVX. Active against *gyrA* mutants. In VAS regimen, >90% eradication success.	[33,36,37,40,41,42]

CDI—*C. difficile* infection; CIP—ciprofloxacin; FQs—fluoroquinolones; LSFX—lascufloxacin; LVX—levofloxacin; MFX—moxifloxacin; MTZ—metronidazole; VAS regimen—vonoprazan, amoxicillin, and sitafloxacin regimen.

**Table 2:** Activity of some newer tetracycline derivatives against anaerobes and *Helicobacter pylori*.

Antibiotics (Abbreviations) Activity Against	*C. difficile*	*Bacteroides* spp.	Other Anaerobes	*H. pylori*	References
**Eravacycline** (**ERV**, a fully synthetic 3rd-generation tetracycline (fluorocycline) antibiotic)	Lower ERV MIC_90_ (0.016 mg/L) than FDX, MTZ and VAN. Bactericidal activity against RT027. ERV does not induce CDI.	ERV MICs_90_ were 2 to 16-fold lower than those of TGC against *Bacteroides/Parabacteroides* spp.	Potent in vitro activity against *Prevotella, Fusobacterium* spp., anaerobic cocci, *P. asaccharolytica*, *C. acnes*, and other anaerobes.	Lower MICs than TET.	[50,51,52,53,54,55,57]
**Omadacycline** (**OMC**, a semisynthetic 3rd-generation tetracycline, aminomethylcycline)	OMC MIC_90_ (0.5 mg/L) slightly higher than that of TGC (0.25 mg/L).	OMC MICs of 0.25–16 mg/L for *B. fragilis*, 0.12–16 mg/L for *B. thetaiotaomicron*, 0.06–2 mg/L for *B. vulgatus* and 0.06– >16 mg/L for *B. ovatus*.	Activity similar to that of ERV, but not as strong in vitro against gram-negative bacteria.	Lower MICs than TET.	[43,44,57]
**Sarecycline** (a third generation and narrow-spectrum derivative of tetracycline)			A narrow-spectrum oral antibiotic with double binding to *C. acnes* ribosome, successful for treating inflammatory non-nodular lesions of moderate/severe acne in patients aged ≥ 9 years.		[58,59,63]

CDI—*C. difficile* infection; ERV—eravacycline; FDX—fidaxomicin; MTZ—metronidazole; OMC—omadacycline; TET—tetracycline; TGC—tigecycline; VAN—vancomycin.

**Table 3:** Activity of other newer antibiotics against anaerobes and *Helicobacter pylori*.

Antibiotics Activity Against	*C. difficile*	*Bacteroides* spp.	Other Anaerobes	*H. pylori*	References
**Amixicile** (nitazoxanide derivative)	Preclinical studies	Preclinical studies	Prevents oral periodontal pathogen overgrowth. Potential candidate for treatment of periodontitis.	Preclinical studies. Cross-resistance with MTZ may limit its use.	[67,69,70,71]
**Beta-lactam/beta-lactamase inhibitor antibiotics:** **aztreonam/avibactam, cefepime/enmetazobactam, imipenem/relebactam, meropenem–vaborbactam**		No improved activity compared to the antibiotic in the combination alone.	No improved activity compared to the antibiotic in the combination alone.		[72,73,75,76]
**Cadazolid** (quinoxolidinone antibiotic). Discontinued studies.	No superiority over VAN. Lower recurrence rate than by VAN.				[77,78]
**CRS3123** (1-benzopyran compound)	Active against *C. difficile*, suppresses its toxin production and sporulation. Lower recurrence rates than by VAN.				[79,80]
**Gepotidacin** (triazaacenaphthylene antibiotic)	MIC_90_ (2 mg/L) was higher than that of MTZ (1 mg/L), but lower than those of CLI, MFX, IPM, and PTZ.	MIC_90_ (4 mg/L) 4-fold higher than those of MTZ and IPM, and 2-fold lower than those of CLI and PTZ.	Against gram-negative anaerobes, MIC_90_ higher than those of MTZ and IPM, but lower than those of CLI and PTZ. MIC_90_ against gram-positive anaerobes, higher than that of MTZ, but lower than those of CLI, IPM, and PTZ.		[81]
**Ibezapolstat** (inhibitor of the PolC bacterial protein)	Non-inferiority to VAN in Phase 2b study. Narrow spectrum, highly concentrated in stool, preserving gut microbiota and bile acid balance.				[83]
**Lefamulin** (a semisynthetic pleuromutilin antibacterial)	Not active. Risk of CDI.	Not active.	Activity against *C. perfringens*, *C. acnes*, *Prevotella*, *Fusobacterium* spp., and GPAC. Single data.		[84,85,86]
**Ridinilazole** (bis-benzimidazole antibiotic) Discontinued studies.	Not superior to VAN regarding SCR but reduces recurrences and preserves the gut microbiota.				[10,87,88,89,90]
**Rifasutenizol** (TNP-2198, a hybrid rifamycin and nitroimidazole antibiotic under investigation)	Activity comparable to that of vancomycin in an animal model.		Potential activity against *Gardnerella* spp.	A 14-day oral triple TNP-2198-based therapy achieved > 90% eradication rates for *H. pylori*.	[91,92,93,94]
**Zoliflodacin** (a spiropyrimidinetrione antibacterial agent)				High in vitro activity against *H. pylori*, MIC range, 0.008–1 mg/L. Potential anti-biofilm and synergism with other antibiotics.	[61,95,96]

CDI—*C. difficile* infection; CLI—clindamycin; IPM—imipenem; GPAC—Gram-positive anaerobic cocci; MFX—moxifloxacin, MTZ—metronidazole; PTZ—piperacillin–tazobactam; SCR—sustained clinical response; VAN—vancomycin.

## Data Availability

Data are contained within the review.

## Abbreviations

The following abbreviations are used in this manuscript:

BFG	*Bacteroides fragilis* group
CDI	*Clostridioides difficile* infections
EMA	The European Medicines Agency
FDA	The U.S. Food and Drug Administration
MDR	Multidrug resistance
MIC	Minimal inhibitory concentration
PFOR	Pyruvate ferredoxin oxidoreductase
SCR	Sustained clinical response
